# A Systematic Review of Objective Factors Influencing Behavior in Public Open Spaces

**DOI:** 10.3389/fpubh.2022.898136

**Published:** 2022-05-06

**Authors:** Shuyan Han, Yu Ye, Yifan Song, Shurui Yan, Feng Shi, Yuhao Zhang, Xiaodong Liu, Hu Du, Dexuan Song

**Affiliations:** ^1^Department of Architecture, College of Architecture and Urban Planning, Tongji University, Shanghai, China; ^2^Ministry of Education Key Laboratory of Ecology and Energy Saving Study of Dense Habitat (Tongji University), Shanghai, China; ^3^Department of Architecture, College of Design and Engineering, National University of Singapore, Singapore, Singapore; ^4^Department of Architecture, School of Architecture, Tsinghua University, Beijing, China; ^5^Department of Architecture, School of Architecture and Civil Engineering, Xiamen University, Xiamen, China; ^6^Department of Architecture, School of Architecture, South China University of Technology, Guangzhou, China; ^7^Welsh School of Architecture, Cardiff University, Cardiff, United Kingdom; ^8^School of Civil Engineering and Built Environment, Liverpool John Moores University, Liverpool, United Kingdom

**Keywords:** outdoor activity, public open spaces, objective influencing factors, behavior-environment interactions, built environment

## Abstract

As a significant part of outdoor built-environment, public open spaces are closely associated with people's daily lives. Studies of outdoor behavior in these spaces can shed light on users' environmental perceptions and contribute to the promotion of physiological and psychological health. Many recent studies are case studies focused where observations, surveys and interviews have been conducted to understand the factors influencing people's behavior on one or few sites or city environments. There have been few reviews related to this topic, and none have been based on the systematic understanding of influencing factors. This paper presents a systematic review of interactions between behavior and the built environment in public open spaces, and highlights the impacts of diverse and objective influencing factors. Followed the rules of PRISMA method (Preferred Reporting Items for Systematic Reviews and Meta-Analyses), 109 papers published in 2000–2021 were selected and reviewed. The distribution of the studied interactions is analyzed, and the impacts of four distinct factors: personal background, location and context, environmental component, and climate stimuli, are extracted, categorized, and specified. Moreover, outdoor health benefits are discussed based on which, crucial factors that require emphasis after the outbreak of COVID-19 are identified. Throughout this paper, behavioral influencing processes, including objective influencing factors, subjective feedback, and the relationships involved, are considered to provide a comprehensive picture. With the robust classification of existing factors, architects, urban designers, policy makers and fellow researches could be easier to get a more comprehensive trend from the past. This paper also provides guidance for future research, especially given that COVID-19 has created huge changes to outdoor needs and customary behavior.

**Systematic Review Registration:** http://www.prisma-statement.org/.

## Introduction

### Background

Public open spaces, as the main component of outdoor environments in urban public lives, refer to freely accessible parks, green spaces, esplanades, plots with sports and leisure facilities, and other spaces that offer opportunities for recreational activities and act as visual amenities (1, 2). Public spaces are strongly associated with people's outdoor recreation activities and daily lives; as such, people's behavior has been highly mentioned in public space-based research.

**Figure 1:**
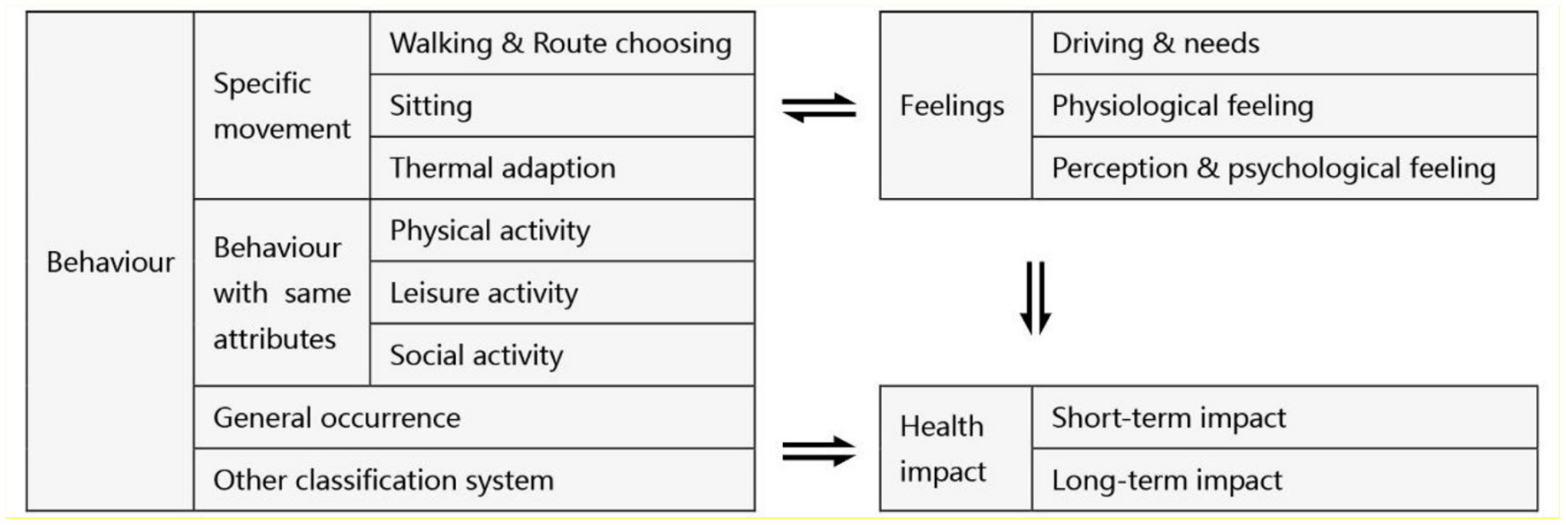
Subjective feedbacks frequently investigated in public open spaces.

A growing number of studies have been conducted to investigate behavior, background situation and information (3), the underlying drive (2), objective and subjective aspects of the influencing factors (4, 5), and behavior-related feedback (1, 6, 7). According to the previous study, the subjective feedback can be divided into three categories: people's behavior, feelings and health impact (see Figure 1).

Due to the focus on multiple types of outdoor factors and diverse behaviors, outdoor-behavior-based investigations are complex. Certain movements such as walking (8, 9), route-choosing (10), sitting, and thermal adaption (11) have been considered as the most common activities. While studied with similar research objects, topics more clearly targeted are physical activity, which refers to any bodily movement produced by skeletal muscles that require energy expenditure (12) closely associated with health impacts (13–18); leisure activity that is more inclined toward outdoor leisure, recreation (19, 20), and short-term recovery; and social activity, mainly studied as social factors closely associated with people's needs. General occurrence, as a reflection of overall behavior without type classification, has also been frequently studied to highlight the impact of identified factors on the premise of the overall environment.

Moreover, people's feelings were considered closely associated with outdoor behavior. Drive and needs, physiological feeling, environmental perception, and psychological feeling, have been frequently mention and studied in exist studies (21–34). Among them, drive and needs, considered as the origin of outdoor behavior, have been studied linked with provided locations. Physiological feeling and thermal comfort, not only associated with thermal adaption (35) but also linked with feelings and emotions (36), were considered could affect places' overall attendance. Moreover, investigations based on environmental perception and psychological feeling were also applied in some researches to measure more in-depth interactions (37).

Health impacts were also investigated in some behavior-based studies, including long-term impacts such as physical and mental health and short-term impacts such as fatigue restoration, attention recovery, and emotion elevation (38–40).

**Figure 2:**
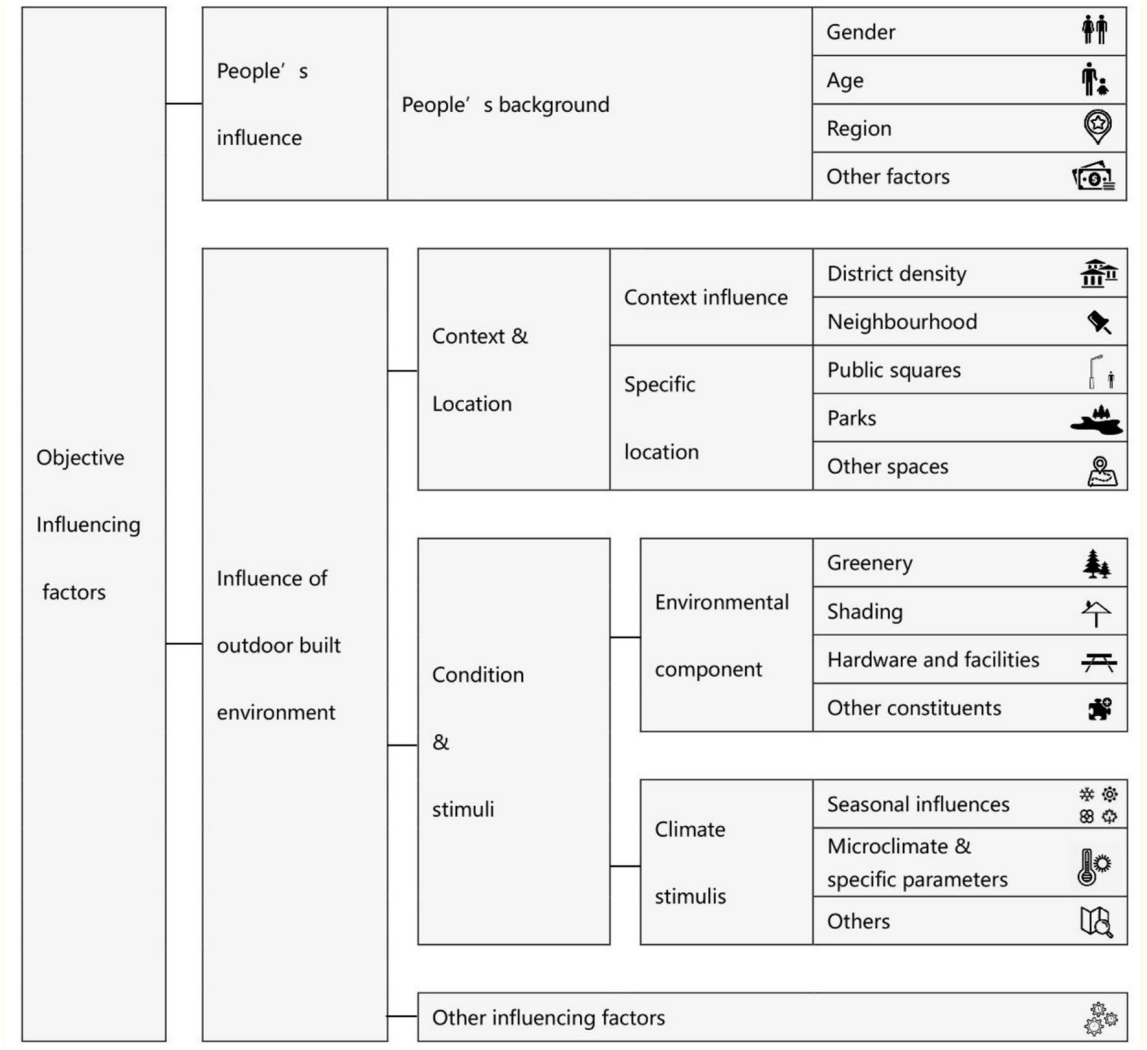
Objective influencing factors frequently investigated in public open spaces.

The objective influencing factors can be divided into five categories (see Figure 2): people's background, the built environment context, environmental components, climate stimuli, and others. Those environmental design related studies focused more on site usage and people's occupancy, whereas medical-based reviews focused more on category, strength, and promotion of outdoor activity associated with health promotion (41). Physical activity and activity with physical attributes (42) were mentioned more often in medical-related reviews. Microclimate was found to have a significant influence on the general occurrence and thermal adaption (43), among which temperature, solar radiation, and wind speed were shown to be the most significant factors (44). Moreover, naturalness, expectations, time of exposure, perceived control, and outdoor experience were found to have an impact (35) as well as personal factors, social factors, perceptions of the built environment, and physical factors (45).

For behavior with physical attributes, reviews conducted were more precise: activities including walking (46) and dog-walking (47); backgrounds including country and region, location, context, gender, and specific groups (41, 48, 49); social factors including social support, networks, cohesion, capital and socioeconomic position, income inequality, and racial discrimination (50), and identified places including parks, neighborhoods, or places with physical facilities (51–53) were focused on and frequently linked with health promotion effects.

### Review Objectives

Existing reviews mostly focused on a particular type of activity or an influencing factor and lacks a comprehensive understanding of the interactions associated. This review fills the research gap by offering a systematic comprehension of influencing factors in outdoor built environment, because outdoor activities were often affected by complex environmental stimuli.

The first objective of this review is to introduce the background, importance, and method of the research topic which are included in Section Introduction and Method of Review. The second objective is to summarize the types of activities studied in previous articles and to establish the association between objective influencing factors and subjective feedbacks. The third objective is to categorize the impact of objective factors, such as people's background, context and location of built environment, environmental component and constituent, the influence of thermal and other climate-based stimuli. The final objective is to understand the changes in outdoor behavior-based studies after the outbreak of COVID-19 pandemic.

## Method of Review

**Figure 3:**
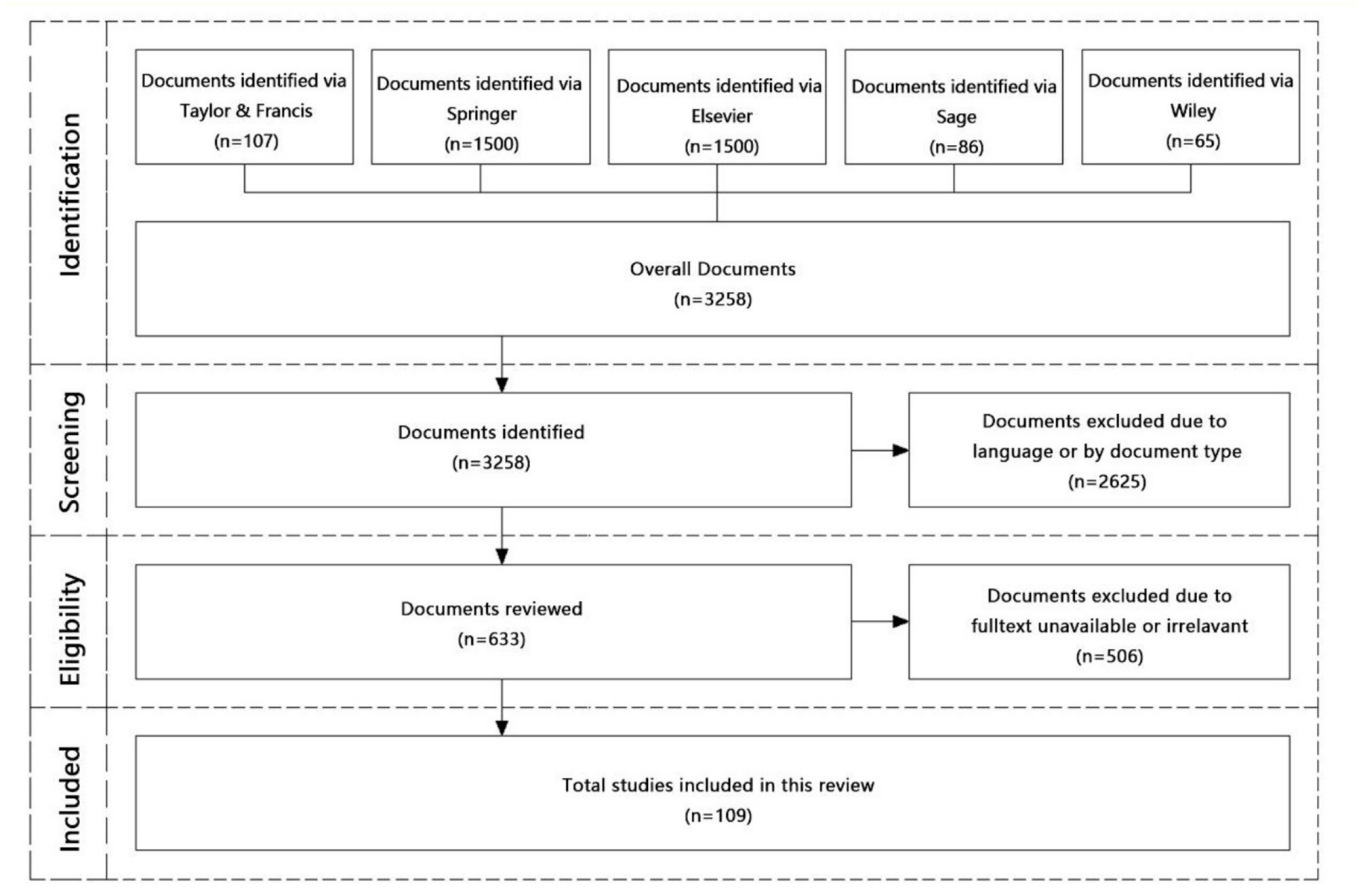
Method of review.

This article focuses on outdoor behavior-based publications from the scope of architecture and urban design. Since the application of quantitative research in this field is mainly concentrated after year 2000. Articles using quantitative methods and published from 2000 to 2021 were focused. Followed the method of PRISMA (Preferred Reporting Items for Systematic Reviews and Meta-Analyses), studies based on behavior investigation in public open spaces were identified as shown in Figure 3 which including following four stages:

(1) Identification: In the field of architecture and urban design, academic journals are commonly published by Elsevier, Springer, Taylor and Francis, Sage, and Wiley. Therefore, related research engines were chosen during identification. Web of science was not considered due to the similar databased and search outputs.

(2) Screening: Using keywords (outdoor OR public OR ‘open space') AND (behavior OR behavior OR activity OR activities), 3,258 papers related to this review topic were downloaded. Precisely, 1,500 papers most related were downloaded from Springer and 1,500 from Elsevier, and they are identified searching through title, abstract, or keywords. Additional 107 papers from Taylor and Francis, 86 papers from Sage, and 65 papers from Wiley were identified using full text research.

(3) Eligibility check: A more specified screening were conducted though full texts check. Articles written in languages other than English were excluded; studies full-text unavailable were removed, investigations only based on outdoor occurrence without activity studied were excluded; and papers using simulation methods without *in-situ* monitoring were not considered.

(4) Inclusion: After selection, remaining 109 articles were included for this systematic review.

## Overview of Identified Papers

### Distribution of the Types of Studied Behavior

**Figure 4:**
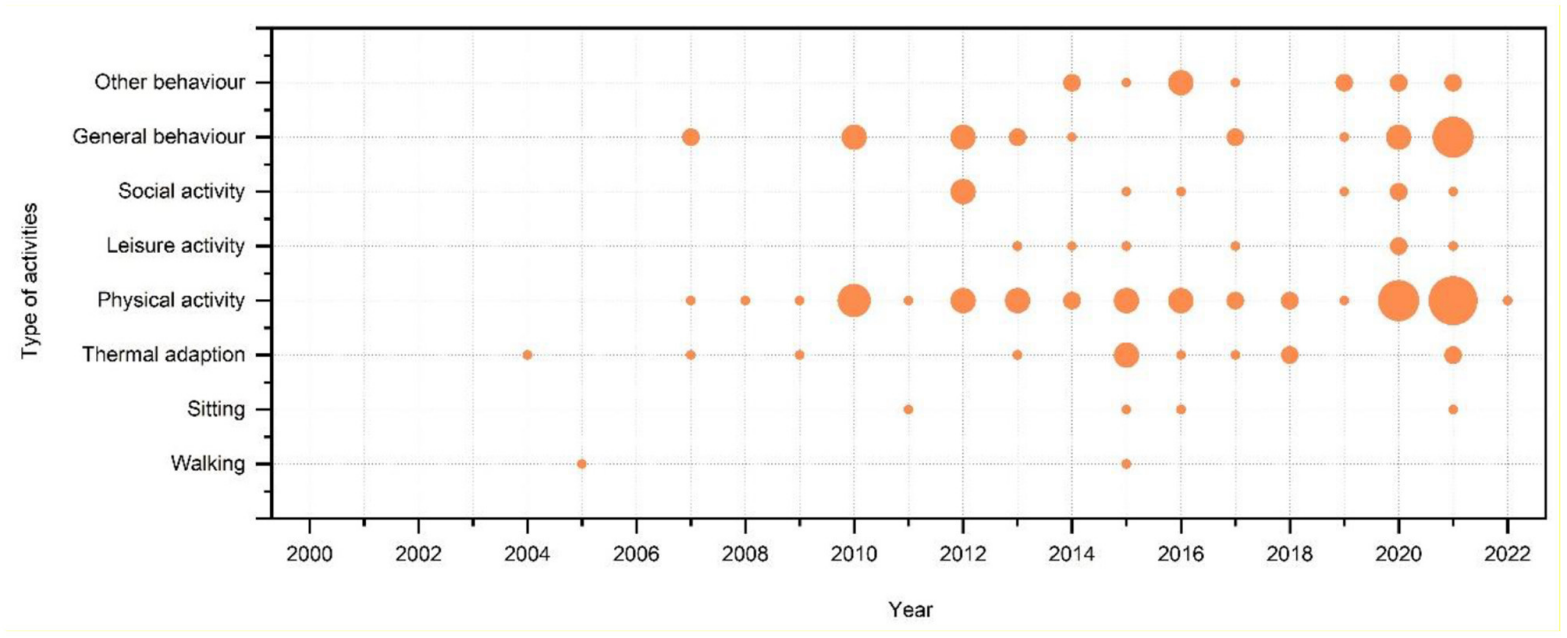
Distribution of types of behaviors being studied or focused.

Based on our sample of reviewed papers, the distribution of the behaviors these studies focused on is shown in Figure 4. Overall, the number of behavior-based investigations has increased gradually over the past few years, and has risen rapidly in 2020 and 2021. Physical activity, and general occurrence were the two major types.

Among all types of behaviors, specific movements have been less studied; only a few studies investigated walking (54, 55) and sitting (56–59). By comparison, the studies focused more on thermal comfort and climate conditions (60–72).

Owing to the complexity of outdoor spaces, specific movements and types of behavior are usually considered to have different attributes; for example, recreational walking with friends can be classified as physical, leisure, and social activities. Regarding the focus of activity attributes, physical activity (73–111), leisure activity (112–118), and social activity (119–127) have gained more attention. Due to the close link with health impact, several of these studies are associated with physical activity, and this stream of research has risen significantly in the past 2 years because of the focus from the field of environmental design.

Mainly related to thermal comfort and space design, general occurrences (36, 128–148) have received continuous attention. Because of the focus on people-centric designs, more investigations have been conducted on this topic since 2014 with the adoption of other types of systems (149–161).

### Distribution of Interactions Between Objective Influencing Factors and Subjective Feedbacks

**Figure 5:**
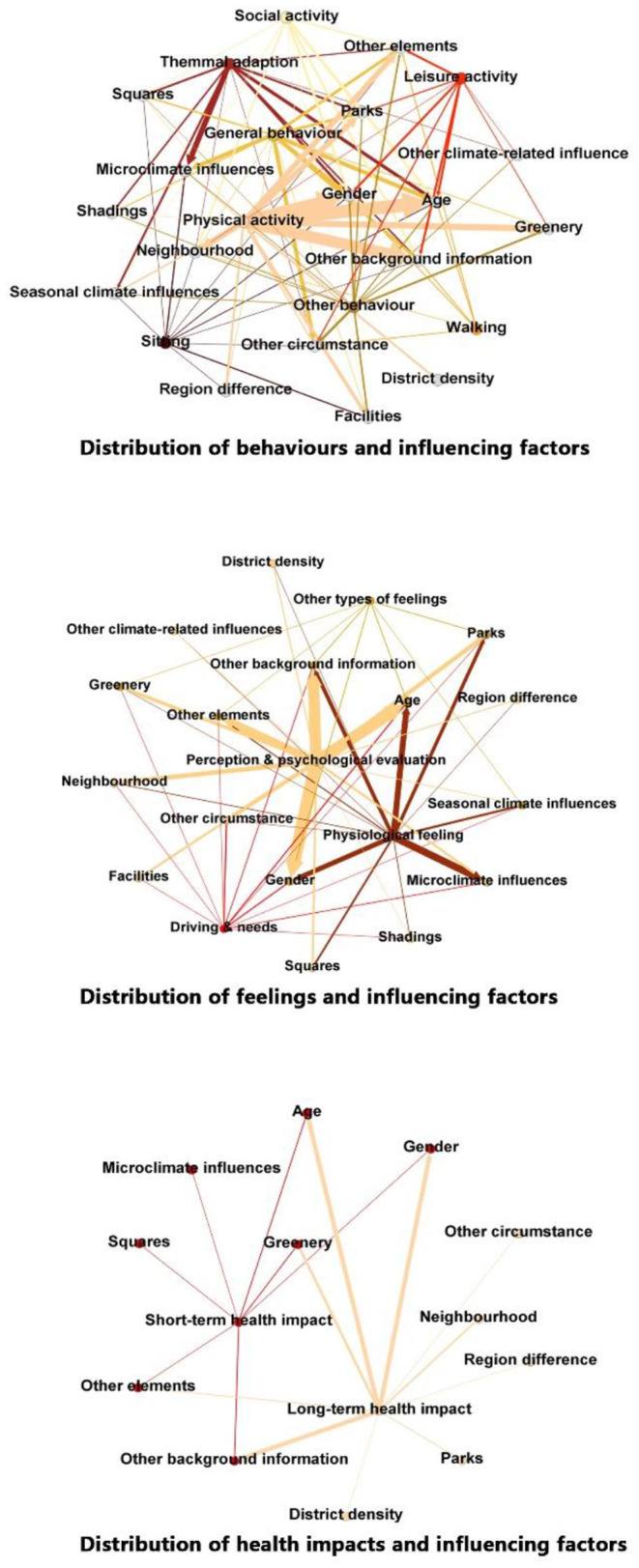
Distribution of objective influencing factors.

The number and distribution of interactions in the studies by subjective feedback and objective influencing factors are shown in Figure 5.

Associated with behavior, great differences can be found in the activity characteristics and research focus. Without mention of utilitarian walking on streets, recreational walking in public open spaces such as neighborhoods and greenways has seldom been studied. In comparison, although sitting is also not frequently shown as a research focus, more investigations have been conducted in which sitting was considered part of outdoor leisure and thermal adaption. For physical activity, leisure activity, and social activity, interactions differed greatly because of behavioral attributes. Because of the wide range of physical activity and the large number of related studies conducted, physical activity investigation was associated with the most influential factors. In contrast to physical activity, leisure activity has shown a prevalence in parks and neighborhoods compared with squares because of environmental recreational attributes. Social factors were studied more as subjective factors that can affect general occurrence in existing research; thus, there was less focus on the influence of identified components, such as greenery and shading.

Among all types of the studied feelings related to behavior investigation, there was less focus on needs and drive, although they are extensively linked with all types of factors because of their association with most behavior. Physiological feelings, mainly associated with thermal adaption and comfort studies, were more related to climate-based influencing factors compared with others. Environmental perception, which is widely associated with all types of influencing factors, was the most studied among all types of feelings.

Although many outdoor behaviors can affect health conditions, only a few studies have directly investigated such interactions. There was a greater focus on people's background factors than thermal-based impacts. Short-term impacts, including fatigue recovery, cognitive ability, feelings, and emotions, have been more studied and linked with greenery. Long-term impacts, including both physical and psychological health, were also highly linked to greenery, and neighborhood greenery has been emphasized.

## Effects of Objective Influencing Factors

**Figure 6:**
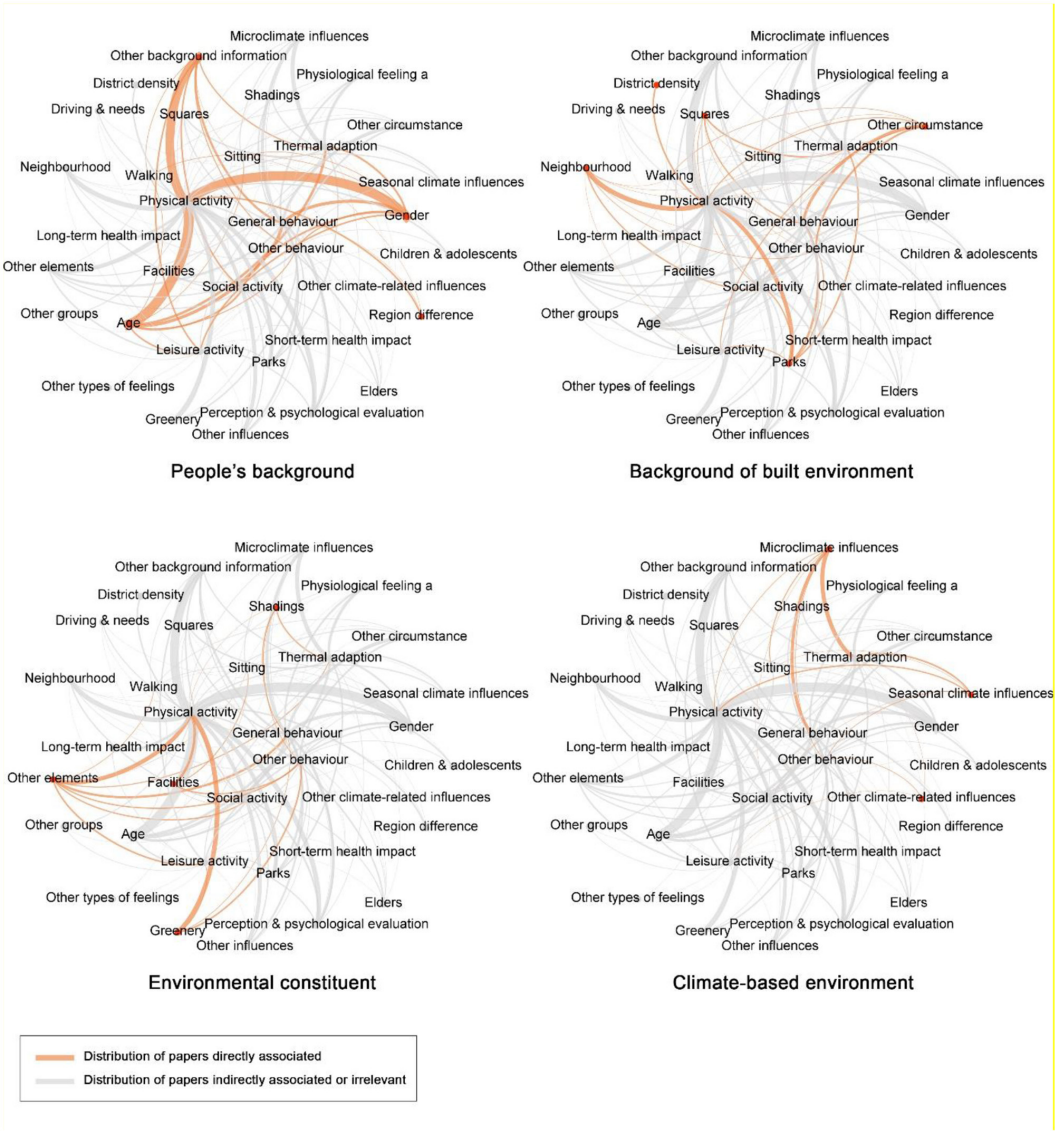
Direct associations related to types of factors.

**Figure 7:**
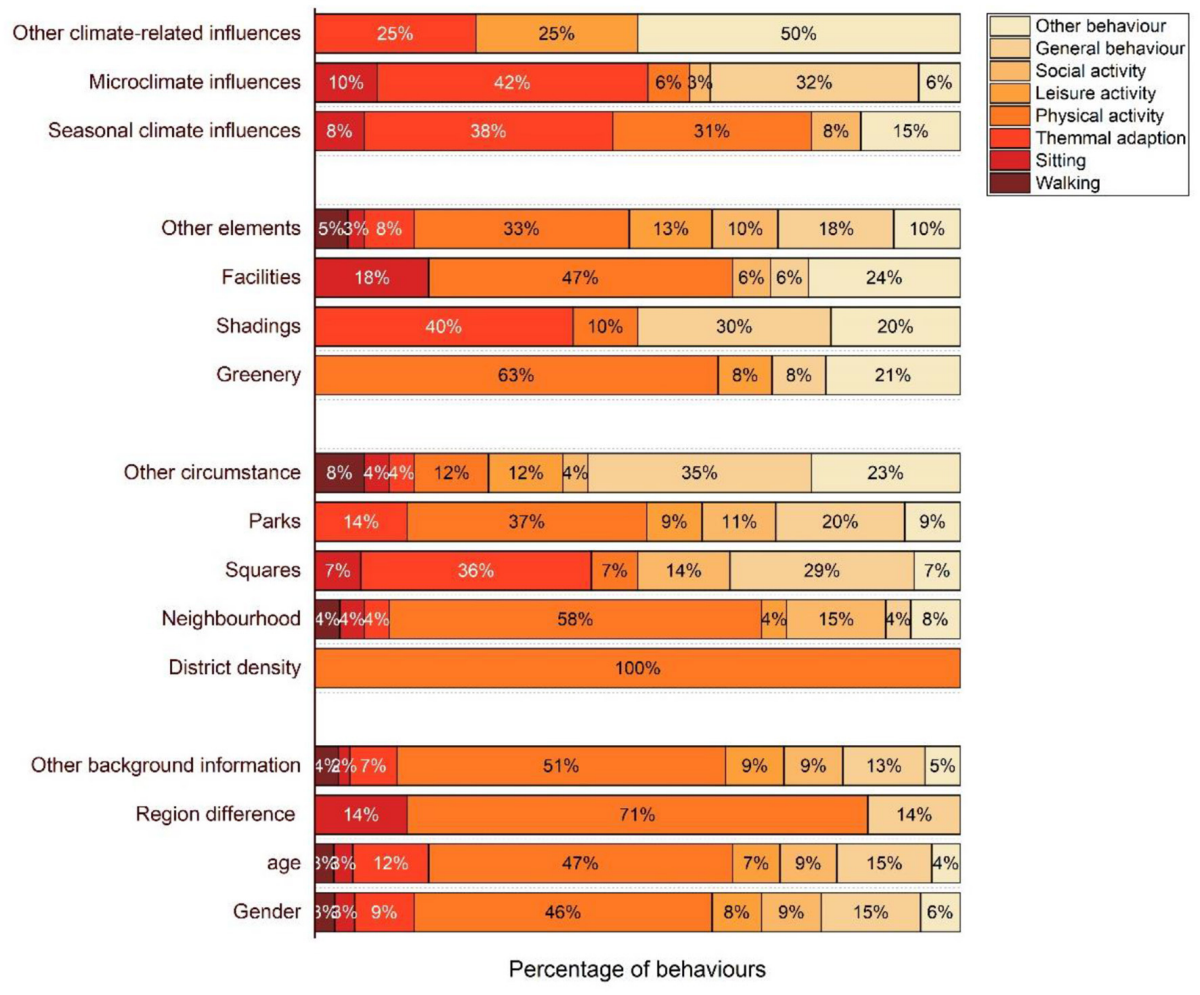
Distribution of behaviors related to types of factors.

Referring to existing research, complex influencing factors and interactions were investigated, as shown in Figure 6. Owing to the factors' characteristics, the type and proportion of associated behavior were diverse, as shown in Figure 7. Specifically, the associated behavior, outstanding conclusions, and focal points are categorized and identified as follows.

### People's Background

In most cases, based on questionnaires, people's backgrounds, including demographic features and socioeconomic status, were considered to affect outdoor behavior greatly. Based on people's age, gender, region, and other background differences, site preference (154), thermal tolerance (157), and psychological feelings (30) were found to have significant differences. Therefore, respondents' backgrounds were well-controlled (104) or adjusted (85) in most behavior-based investigations.

#### Gender

Gender is one of the main factors that can affect people's outdoor attendance, preferences, and feelings. Besides physiological and psychological differences, gender-based differences can be inferred through acquired gender patterns (78) and traditional gender roles in some places. For example, only elderly females showed higher levels of activity and less sedentary behavior in the presence of higher retail, food outlet, and public transit densities at short distances. By contrast, only elderly males showed significant associations with entertainment density, light-to-vigorous physical activity, and sedentary behavior (93).

In terms of overall attendance and preference, males were found to be more physically active, spend less time at home (87), and be more enthusiastic about physical-related features and facilities. More males than females were observed using parks, and males were twice as likely to be vigorously active (105). Such tendencies were also found in elderly groups: elderly males were more active in parks than females, although fitness and health-related physical activity such as walking and fitness exercising were found to be especially attractive to females (78). For younger adults, recreational facilities such as greenery were found to be associated with physical activity measures for females (87) while males were found to care more about sports features (94). Moreover, fitness equipment and sports courts were found to be positively correlated with engaging in physical activity for male adults (91).

Psychological feelings and the need for spaces were also found to differ, among which safety was one of the main attributes. For urban parks, safety was assessed as satisfactory for all genders, while female respondents were found to have greater security requirements. For the same outdoor environment, female respondents were found to be significantly less satisfied than male respondents (70). For adolescents, boys were more concerned about characteristic features and environmental design, while girls were more concerned about accessibility and safety (95).

In addition, differences can be found regarding the natural environment. Males were found to be more sensitive to environmental change, and in an investigation of the dose-response curve between stress reduction and natural exposure, only males were found to have an inverted-U curve shape with varying densities of tree cover and stress recovery (40). Meanwhile, more positive feedback was found in females: girls were more attracted to playing with water than males in urban green spaces (114). Similar differences were also found in thermal aspects: women were more positive when responding to an uncomfortable environment. And female pedestrians were also found more careful about protecting themselves from solar radiation than males in subtropical area (11).

#### Age Difference

Participants' activities, habits (152), preferences (114), concerns (95), and emotional estimations of outdoor urban places (27) were diverse because of age differences.

In an open space, people of different ages have diverse preferences for use. Design elements in open spaces were found to be linked to activities, and young children were identified as mostly using playgrounds and natural lawn areas. Older school children and teenagers tended to use sports areas and semi-secluded spaces to play sports or to meet in groups and socialize. Meanwhile, older peoples' sedentary activity was exclusively observed on benches, and non-sedentary activities such as jogging were nearly exclusively identified in larger, more shaded parks (86). Identified environmental factors were found to be related to activities of different ages. Associations between greenness and physical activity for individuals were highly linked with age, especially in people aged 20 to 30 years, attributable to their special life stage. However, the investigation did not demonstrate a strong association between levels of physical activity and greenness among older adults, which was conjectured to be caused by their health condition and limited mobility (87).

#### Regional Differences

Discrepancies caused by regional differences are diverse: race, culture, thermal environment, different experiences, and even regional economic situations (80) can produce different outdoor feelings, perceptions, and habits. Thermal preferences and behavioral habits are the most studied topics associated with the design of public open spaces.

Climate-based environmental perceptions, adaptation, and attendance can differ. Environmental tolerance was surveyed in a fixed location to study people's perceptions (32), among which minor tolerance was found in local respondents compared with tourists, especially those from denser and more polluted cities. Furthermore, the thermal feeling is strongly linked to the climate zone of habitation: the thermal comfort range and neutral temperature of subjects in hot and humid regions were found to be higher than those of people in temperate regions (72). Under similar thermal conditions, the Japanese evaluated the weather as warmer and less preferred for outdoor activities, while the Swedes felt happier, calm, and pleasant, which tentatively indicated a difference in environmental attitudes between people from different countries (34, 37).

In addition, cultural and social backgrounds can cause behavioral differences. Socioeconomic and cultural influences were compared in North Africa and America under the same climate conditions (56), and it was found that a higher number of people in North Africa tended to spend more time outdoors and with larger groups or families, but with less variety in activities carried out. Behavior types were also found to be different; people in North Africa tended to sit more and wear more clothes (56). Furthermore, recreational walking behavior was compared between neighborhoods in 12 countries, and it was found that participants in some European countries tended to walk more often and longer for recreation and reported better environmental perceptions compared with people in Latin American countries that reported lower walking frequency, duration, and some environmental attributes (33). Moreover, the difference in outdoor behavior can also be based on different city density compositions and traditions: walking behavior was tested and reported to be different in Brisbane and Hong Kong. Specifically, older adults reported better access to most destinations and accumulated significantly more minutes of walking, particularly for shorter distances in Hong Kong because of differences in accessibility and city density (25).

#### Other Factors

Health, social, economic, and educational status were also found to be related to outdoor behavior. In an investigation of hot arid climate zones, researchers found that the upper band of people tends to spend less time outdoors (56). In America education was found to exacerbate the negative effects of adverse weather conditions on the decision to exercise outdoors, while another investigation found that people with higher education levels were more likely to walk and exercise outside, and those of lower socioeconomic status were more affected by outdoor weather (85). In an investigation based on objectively assessed neighborhood characteristics (93), social factors rather than physical capacity and health status were found to influence older adults' total physical activity more strongly. Compared with age and health-related status, education and car ownership were found to be more strongly associated with physical activity and sedentary behavior (93). Furthermore, as the elderly have functional limitations and perceived problems, type of limitation was found to affect people's behaviors. People with both movement and perception or cognition-related functional limitations were less satisfied with the frequency of activity, which may be because of the negative health experience and problems in the pedestrian environment (22).

### Background of Built Environment

The location of the studied public open spaces is diverse and can be analyzed based on several aspects: (1) the influence of cities or districts, such as district density (36, 141) and neighborhoods; (2) identified location being investigated, such as parks (9), squares, and other open spaces; (3) other characteristics of locations associated with the research focus, such as investigation of shopping that emphasizes the commercial background (4); and the study focused on seniors' behaviors that highlights old city zones (149).

#### The Influence of District Density

City density is highly related to people's behavior and needs. Better accessibility and perceived accessibility to destinations, including services and public transportation, are deemed to facilitate an active lifestyle (93). Such an impact is particularly clear for older adults: higher accessibility to most destinations could accumulate significantly more minutes of walking for the elder (25), which may partly explain why older adults living in Chinese and other high-density Asian cities accumulate substantially more physical activity and less sitting time than their Western counterparts (93). The need for infrastructure may also differ owing to density discrepancy. Elderly residents living in suburban areas need to feel secure when accessing close-to-home green spaces, while elderly people living in urban residential areas need more connection to green spaces and frequent physical activity in association with commuting (100). Moreover, people's perceptions can also differ. People living in rural areas are more sensitive to outdoor environmental changes such as seasonality impacts (162) compared with those living in urban areas. Moreover, people living in high-density residential environments require more natural scenery than low-density built forms (129). For the reasons mentioned above, the research conducted slightly differed: more research focused on urban-based public open spaces, including studies of thermal perception and experience (63, 140), restorative processes (38), and health-based investigations (29, 101, 109).

#### The Influence of Neighborhoods

Neighborhoods are closely associated with daily life. Unlike public open spaces far away from home, they usually link activities with noted purposes. Public open spaces in neighborhoods or residential areas can provide easily accessible places for physical and leisure activities and play an important role for residents with functional limitations. Because of a higher frequency of use and better accessibility for residents, studies based on neighborhoods are highly linked with people's health situations, especially for the elderly.

To study the behavior in neighborhoods, common research contents such as specific constituents (74, 87), outdoor comfort parameters (64) perceived environmental attributes (23, 33), and other aspects were taken into consideration. Utilitarian destinations of neighborhoods such as grocery stores, restaurants, retail stores, and convenience stores, were found to be significantly correlated with walking and physical activities. Housing type, sports facilities, and transportation infrastructure were found to be associated with vigorous physical activity (98). Because of fear of crime freedom has been restricted, preventing residents from engaging in outdoor activities (123). In addition, environmental stressors, including neighborhood incivilities, traffic noise annoyance, and air pollution concerns related to neighborhood aesthetics ratings, were taken into consideration (23).

Closely associated with leisure-time physical activity (81), residents of advantaged neighborhoods reported significantly higher levels of total activity and were less likely to walk for transport (75). Because of the close link with physical activity, people's health is one of the main focuses in neighborhoods. Close-to-home green spaces were spotlighted and proven to elevate the rate of physical activity, which was further linked to self-rated health (100, 110). Among the existing studies, stress mitigation (103), mental health (111), and physiological health including excess weight, obesity (110), and cardiovascular disease (111) were all considered.

Elders were particularly focused on the neighborhood because of functional decline and inactivity. Physical and social environments were considered to affect the elderly's physical activity (79) and were further associated with their ages (104). Among all types of influencing factors, the mean radiant temperature, air temperature, greenery, and outdoor seating were found to correlate with space usage (57). Parks located near homes served as a place that can provide social interaction (122), which was found to have a greater influence on physical activity than physical capacity and health status (93). Moreover, other factors including safety, aesthetics, amenities, cleanliness, comfort, density, urban landscape (21), and the elderly's personal information such as gender, age, education, car ownership, and health-related status (93) were also considered. Neighborhood environments are increasingly salient to the elderly who face multiple personal and social changes that often limit their daily activities to their immediate or nearby surroundings (79).

#### Squares

Because of the low interference of plants and greenness, the research conditions in squares are relatively simple. Research has mainly focused on changes in specified influencing factors or activity. Plaza users' behaviors, including activity type, attendance, distribution, and activity level; the influence of physical factors, including environmental conditions, shading, temporary design, and thermal comfort (62, 67, 72, 142, 148, 155); subjective perception such as the width of the square, spatial openness, and appearance of materials (140), as well as other influencing factors such as culture and environmental attitude (37), threshold density, and sheltered seating (58) were taken into consideration. In general, square-based studies were less interfered with compared with other types of public open spaces and focused more on the identified behavior without the influence of greenery and health-related factors. Therefore, thermal adaption and general occurrence were more focused on and studied in public open squares.

#### Parks

Because of their close link with leisure, recreation, and physical activity (52), parks are the most frequently studied form of public outdoor space (19, 60, 65, 130). Based on a wide range of park-based investigations, types of behavior, influencing factors, and diverse background locations, including neighborhoods, residential areas, hospitals, and city centers, have been studied (92, 145). The accessibility of parks has been highlighted in existing research: visitors to nearby, medium-distance, and distant parks vary significantly in terms of park visit frequency, travel mode, time spent in parks, and type of activity (133). The maximum number of park visits dropped exponentially as the travel distance to parks increased (133) while perceived accessibility was found to have an even stronger explanatory power (135).

Park use was significantly higher among dog walkers, couples living with children, and those living in the least disadvantaged areas (107) and was highly associated with physical activity and leisure activity (106, 153). Park users were found to be more active than non-users in all forms of physical activity except for transport walking (101), and were more likely to meet the physical activity guidelines than those who indicated that they did not regularly use a park (107). Among all age groups, the elderly's use of parks has been focused on (86). Seniors visited urban parks frequently and spent more than half of their visit time engaging in moderate to vigorous physical activity, especially for males younger than 70 years old and in good health condition (83). Regarding the promotion of activities, park usage was found to mitigate stress by fostering social support (103) and benefits in terms of relaxation, self-perceived confidence, and mental health (101), which were further associated with environmental, social, and psychological health benefits (108).

Particularly, the characteristics, components, and conditions of parks were found to be influential. Physical activities conducted were found to differ by park type. Linear parks were found to provide the greatest overall physical benefit, while other park types provide nonphysical community benefits (108). The size of the park was also found to be positively associated with physical activity participation. Users of larger-sized parks were found to spend more time doing vigorous activities (107) although pocket parks, when perceived as attractive and safe destinations, were also found to increase physical activity by encouraging families with children to walk there (82). Among all types of components, greenness was found to be especially important. The density and quality of greenery can affect people's sensation and satisfaction, which further impacts usage patterns (129, 161). Moreover, the feeling of nature was found to be related to the usage frequency and activity conducted (70). Different parts and components of parks, such as trails, fitness stations, secure areas, lawns, sports fields, and playgrounds, were found to be preferred by different age groups, related to types of activities, and play different roles (78, 83). Moreover, the attendance and use of parks were found to be influenced by thermal conditions (60, 63, 139).

#### Other Open Spaces

Besides the aforementioned sites, existing research has focused on other types of built environments. Among them, places frequently used in daily lives such as nursing homes (144), workplaces (147), preschool centers (90, 158), schools (3), and campuses (113); places with aesthetic values or specific meanings in urban lives such as riverbanks, water features, coastal and marine areas (114, 146, 154); and places used by mixed groups of people such as urban transit areas (32) have been emphasized and studied. To compare the discrepancies among sites, some studies investigated more than one type of built environment (36).

Places renovated have been shown in several studies. Behavior changed after design interventions in urban brownfield (151) and playground (157). Previous living experiences, established social networks, and people's needs were found to play important roles in the renovation of elderly community urban districts (122).

### Environmental Constituent

Environmental constituents, which are highly associated with environmental perception and evaluation, have been the focus of research because of their significant impact on outdoor behavior. Among all the types of environmental components, greenery, facilities, and shading were the most frequently studied.

#### Greenery

Greenery's impact has been mentioned from two perspectives: the constituents of vegetation and the overall influence of urban green space (132), which refers to accessible open spaces with a high degree of vegetation cover (132). Closely related to usage rate, activity pattern, and preference, and indirectly linked with health impacts (42, 73), greenery has been studied in relation to types of behaviors and diverse user groups (77, 87, 95).

Greenery has a direct effect on people's behavior. Typically, such influence is put into effect through changes in people's perceptions, including thermal comfort, preferences, and psychological feelings. The level of space enclosure and greenery density were found to be significantly associated with outdoor microclimate conditions and use behaviors, including visit patterns, sensations, and healing evaluations in dense urban contexts (129). This difference in greenery can influence people's choices, satisfaction levels, and overall perceptions and activities.

The constituents of greenery were found to be associated with diverse behavioral impacts. Overall attributes, including perceived culture, serenity, lushness, plant richness, distribution, density, vegetation quality, design, and maintenance were found to have a significant effect on environmental evaluation (73, 74, 117, 159). Different types of greenery, such as tree canopy and open grass, were found to have different impacts on physical activity intensity (81), and the green coverage ratio and diversity of shrubs were found to be positively related to diverse types of physical activity (89). Additionally, to study outdoor preferences, restorative constituents in urban parks were compared for people who were fatigued and looking for a place to rest. Greenery, such as grass and trees, was found to be more important than decorative components, such as flowers and water features, in attracting respondents (38).

Overall, greenery exposure was found to be positively related to outdoor physical activity (7, 102) and further linked to health impacts. The quality of urban green spaces, self-reported frequency, duration of physical activity, stress, mental health, and physical health were examined (109) and such associations were highly linked to the location of greenness. Meanwhile, the accessibility of green spaces has been emphasized in many studies (110) although it is not a limiting factor for most people (131), and a large number of studies have focused on near-home greenness in residential areas or neighborhoods (29, 40, 74, 81, 100, 103, 110, 111). By comparison, few studies have focused on other areas, such as independent greenness, without the location description (112, 156). In general, physical health, including cardiovascular disease (111) and obesity (110); mental health, including the restoration process (39, 112); stress mitigation (40, 103); positive emotion (112), and other psychological influences (29, 111, 156) were found to be related.

#### Shelters and Shaded Areas

Shaded areas are one of the top influencing factors in people's outdoor space usage (145) and adaption behavior (60). Seasons, climate zones, temperature, and other thermal parameters were frequently mentioned as preconditions in these studies because of the close connection between the preferences for shaded areas and thermal comfort.

People's behavior is highly linked to seasonal thermal changes. In unshaded areas within parks, the number of visitors increases following rising thermal conditions during cool seasons, whereas the number of visitors decreases during hot seasons (60). During the heat stress period, the tree canopy, shaded areas, and water features were found to be the most attractive for outdoor participants (71). In a study based on renovation, shaded shelters and vegetation cover were added in a playground and had a huge impact on people's behavior. More occupants were attracted in summertime; people stayed longer, reported less heat stress, and interacted with each other more often (157). People's preferences also changed in other seasons. During summer, visitors preferred to sit in shaded areas; during autumn and winter, sunlit areas were more popular (61). However, such rules cannot be applied arbitrarily in all situations. The overall attendance and the regional climate need to be taken into consideration. For example, the number of visitors to shaded areas increased with rising thermal conditions in both cool and hot seasons in hot and humid areas (60).

Extreme weather conditions have also been highlighted in some cases. Attendance at the exposed site was found at half of the tree-shaded sites because of the shorter thermally acceptable time in humid-subtropical Hong Kong (66). Under similar climatic conditions, people in Taiwan preferred outdoors shelters (72) and tended to engage in static activities in environments with sufficient shade. Shaded areas were found to be conducive to static activities as the summer became hotter (62).

#### Other Facilities

As one of the most common components of outdoor spaces, seats are frequently used, such as in large-scale public open spaces. Regular and auxiliary seats accounted for more than half of the stay-in activities and nearly one-third of all activities (150). Therefore, the usage, features, and preferences of seats have been investigated (59). Among all studies conducted, elderly have been highlighted. The importance, as well as the number and quality, of seats were found to affect the elderly's usage, preference, and activity (57, 121, 149).

Large-scale sports and living facilities, such as outdoor fitness equipment, gym equipment, coffee shops, and public toilets, as well as walking paths, water features, wildlife, amenities, and dog-related facilities (52, 55, 117, 148, 150) can affect space usage. In public spaces, the facilities and locations that were reported as frequently used differed with the facilities that people preferred and associated with well-being (136). The influence of facilities by user group has been emphasized in some studies. For example, although restricted to male adults, the installation of fitness equipment and sports courts was found to provide opportunities to engage in physical activity (91). In addition to gender differences, age groups were distinct in most investigations. Facilities were highly associated with children's outdoor physical activity intensity. Regarding park usage, children were found to be attracted to parks with sports facilities, playground equipment, toilets, drinking fountains, BBQ facilities, large lawns, and landscaping (86, 96). In schoolyards and preschool centers, facilities were found to affect children's physical intensity strongly (158). Different types of behavior settings, including pathways, play structures, and open areas, can affect children's physical activity level. Moreover, facilities with different attributes, including circular vs. straight pathways; open areas with different ground surfaces; asphalt, compacted soil, woodchips, and sand were also found to play different roles (90). For adolescents, playing sports, meeting in groups, and socializing were found to be the most frequently conducted activities (86), and not only physical activity but also social interactions have been highlighted. Grassy open spaces were important for all activities (127) while facilities including sports courts, fitness equipment, walking and cycling paths, playgrounds, and green open spaces were found to be associated with physical activity. Facilities including barbecue and picnic areas, sports features, seating, shade, and cafés were found to be well associated with social activity (94, 127). By contrast, seniors were found to prefer pathways, paved open spaces, and other natural areas, although they are all less likely to be linked to light or moderately vigorous instantaneous activity (83).

### Climate-Based Environment

Unlike indoor behavior, which is less affected by the overall environment and climate, outdoor behaviors are highly associated with climate situations, among which both objective and subjective influencing processes should be considered. Based on the same outdoor conditions, people's thermal feelings can differ due to individual, physical, social, and psychological differences (37, 57, 68, 119, 143). Psychological impacts of one's perception of environmental features were found to affect thermal comfort and acceptability. For example, thermal comfort in summer can be improved if more trees, water ponds, or shade provisions are perceived to be present in parks (31). Objective parameters, including thermal environment, thermal-related parameters, thermal comfort, and other factors such as air quality, acoustic environment, and other climate-based factors were highly associated with environmental assessments, place perceptions, types of behavior, and place-related attendance (36, 60, 85, 138). These factors were also found to influence the functions of places such as parks and squares (139), particular settings (84), and environmental constituents such as shading (67). And seasonal-based change, microclimate influence, and specific parameters are the most frequently studied influencing factors.

#### Seasonal Changes

Outdoor behavior differs according to seasonal changes, especially in summer and winter (142, 163). Outdoor parameters were closely associated with the season and further linked to people's behavior. For example, during cool seasons, a positive correlation was found among the air temperature, mean radiant temperature, and physiologically equivalent temperature of shaded areas and the number of visitors. During hot seasons, temperatures that reflected solar radiation conditions were found to be negatively correlated with the number of visitors in hot-humid regions (72, 141).

Seasonal influences were associated with the choice of positions. During summer, visitors preferred to sit in shaded areas and adapted their park use behavior by coming later in the evening (70), whereas during autumn and winter, sunlit areas were more popular (61). People's leisure patterns and heat tolerance were also found to change during cool and hot seasons (62). In the investigation of exercise habits, people delayed exercise both in summer and winter, and individuals that listed rain as the predominant adverse weather condition had higher odds of exercising indoors and higher odds of delaying exercise compared with those mentioning heat as the predominant adverse condition. Individuals for whom ice or snow was an adverse winter weather condition were more likely to delay exercise than those who were concerned with the cold weather (85). The usage pattern in outdoor spaces was also found to differ in an educational precinct, and seasonal changes were found to coincide with the type of visitors, frequency of visits, length of stay, category of activities, thermal adaptive measures, and the number of people attending outdoor spaces. Among the seasons studied, the number of people attending the study site was more evidently correlated to micrometeorological conditions in autumn than that in the other two seasons because of the occurrence of cool to cold windy weather conditions (69). Aside from the students, such differences also occurred in the elderly, and the factors influencing the elderly's thermal perceptions varied in the winter and summer. A significant association between thermal acceptability and satisfaction was found with open space (119).

#### Microclimate or Specific Outdoor Thermal Parameters

The correlations between space usage and microclimate indicators such as shade, air temperature, solar radiation, wind speed, and relative humidity have been widely studied (36, 61, 129, 143). Outdoor place–human relationships, including weather parameters (air temperature, wind, and cloudlessness) and personal factors, have been reported to have a significant impact on participants' perceptual and emotional estimations. Among these factors, solar radiation was found to affect the outdoor activity choices of citizens significantly. A significant correlation was found between space utilization and solar radiation conditions, with fewer people visiting the park during summer (141) or experiencing outdoor heat stress (71). In addition to sunlight, thermal conditions have also been associated with overall attendance (130). In some cases, temperature was found to be the most powerful factor influencing space use. This demonstrated that people presented in an open urban space are the most subjected to the air temperature they are exposed to (142). Although great variation in the level of use among spaces cannot be explained solely in terms of microclimatic differences, the use of spaces varies chiefly as a function of microclimate (155). The number of counted park users, particularly children and elderly people, significantly declined at the highest temperature of approximately 30°C (86). The type of temperature was highly linked to behavior. Specifically, a strong link was found between cycling and cold temperature, and between kayaking, and hot temperature (19). To obtain a more comprehensive overview of the outdoor microclimate, some parameters such as UTCI (Universal Thermal Climate Index) and PET (Physiological Equivalent Temperature) were used. Steady-state models, such as the PMV (Predicted Mean Vote) index, may not be appropriate for the assessment of short-term outdoor thermal comfort, mainly because they are unable to analyse transient exposure (63). UTCI was chosen in many studies and was found to affect respondents' feelings, attendance, and choice significantly (11, 66, 128). Moreover, such behavioral responses to the thermal environment were found to differ by gender, age, and types of activities (157). Similar to UTCI, PET has also been used to define outdoor thermal comfort in many studies (34, 141, 142). A PET within a comfortable range was chosen to define thermal comfort conditions, which were further used as a steady background to study participants' thermal, emotional, and perceptual assessments (34).

#### Other Climate-Related Factors

Air conditions are one of the basic influencing factors of outdoor behavior. Air pollution has been found to be highly linked to special kinds of behaviors (19) and is mentioned as one of the environmental stressors proven to influence individual emotional reactions (29). Moreover, auditory and visual elements were also found to influence individual emotional reactions (24, 29, 32).

### Other Influencing Factors

Access to green spaces, parks, and identified facilities has been highlighted in many outdoor behavior-related studies. Among them, accessibility and perceived accessibility were both measured and considered to have high similarity, although perceived accessibility was found to have stronger explanatory and predictive power in some cases (135). Investigations based on accessibility and perceived accessibility were mostly focused on the distance between recreational elements and places, such as parks and green spaces, and were highly linked to people's physical activity, leisure activity, and recreational walking. Perceived accessibility and people's demand had the highest impact on park usage (135). In neighborhoods, only the number of parks within 500 m was found to be associated with physical activity (101) although they were mostly not used by almost half of respondents. Whether respondents used their nearest green space depends primarily on area size, distance to the area, and factors that suggest reduced mobility, such as old age, young children, and poor health (132). Increasing accessibility can help in achieving physical activity recommendations, reduce the possibility of being overweight and obese (110), and restore visitors' moods and energy levels. Interaction with nature was found to bring mental health benefits in terms of relaxation and self-perceived confidence (101).

People were also regarded as influential factors. Large-scale populations, population structure, diversification of rural production and labor patterns, and rural leisure lifestyles were found to influence people's behaviors and routines (163). Perceptions of positive social relationships were associated with greater involvement in leisure activities and with better health, especially in the elderly (118). The effect of privatization of public open spaces on the quality of life of people was investigated, with many studies showing degradation of public spaces' openness to the public as a result of privatization (120). In a study of plaza user behavior, certain social behaviors, such as the impact of smokers and crowding, were examined. Surprisingly, smoking was found to have no effect on the distribution of users and activities. People can accept slightly higher levels of crowding in their preferred condition when environmental conditions are limited. When the threshold density of people was reached, users opted for less ideal conditions, moving into the preferred condition when space became available (58).

Other factors were also investigated. Time's influence has been noted in some studies, such as behavior and activities at night time (116, 125, 126). Constructions, especially perceived neighborhood environmental factors such as street pattern and connectivity, residential density, and land-use mix were found to be curvilinearly associated with walking for recreation (33).

## Discussion

### Outdoor Health Impact

Outdoor activity is considered an intermediate factor between the outdoor built environment and health effects.

#### Associated Behavior

In public open spaces, behaviors such as visit pattern, duration, and sensation were found to be associated with health impact and healing evaluation (129).

Outdoor exposure was found associated with emotion elevation and individual recovery process (112). Air pollution, trash, traffic-related noise considered as environmental stressors (29) and the influence of green spaces were frequently considered in relate studies.

The impact of physical activity in outdoor public spaces has also been highlighted. The World Health Organization has pointed out that every move counts toward better health (164). In green spaces, the health benefits of walking were found to be better than those of sitting (156). The frequency and duration of physical activity were found to be linked with the health situation, including physiological health, such as being overweight or obese (110), and mental health, including restoration, energy levels, relaxation, and self-perceived confidence (101).

In addition, social support and interferences in neighborhoods or open spaces were also mentioned (103) to be associated with stress and health benefits.

#### Associated Factors

The health impacts of greenery have also been emphasized (42, 117). Among them, the density of green spaces is crucial (129). Because of the promotion of physical activity, greener neighborhoods were found to be positively related to physiological health (111). Furthermore, tree density was found to be helpful for the stress recovery process (40). When specific behavior was matched with the appropriate greenery of per capita area, walking in a high per capita area and sitting in a low per capita area were found to have the most beneficial effects with regard to reducing stress, improving mood, and matching the participants' preferences for urban green spaces (156). Moreover, studies on the quality of greenery also examined stress mitigation, levels of well-being, place attachment, and people's attention (29, 39, 109). Moreover, discrepancies can also result from the greenness component and structural difference. Defined as structural components, grass and trees, compared with decorative components, were more valued by people who were fatigued (38).

Neighborhood greenness was spotlighted because of higher exposure rates in people's daily lives. Neighborhood parks were found to promote physical activity directly and mitigate stress indirectly *via* spaces' positive impact on social support (103). The frequency of green space usage was found to decline with increasing accessibility, and respondents living closer to green space were found to be more likely to achieve physical activity recommendations and less likely to be overweight or obese (110). As a result, the greenest neighborhoods were found to have the lowest risks of poor mental health, and cardiovascular disease risk was reduced in all neighborhoods with more than 15% green space availability (111). In addition, the quality of neighborhood green space was found to be crucial. The index score of area-aggregated perceived green neighborhood qualities, including five distinct characteristics of the green neighborhood environment, namely, cultural, serene, lush, spacious, and wild, was proposed and applied, and turned out to have an impact on general health, mediated by physical activity and neighborhood satisfaction (74).

### Scope of Study During COVID-19 Pandemic

Since the outbreak of COVID-19, diversified measures have been adopted to reduce the spread of the virus. Among them, lockdowns, social distance limitations, and public space shutters can significantly affect people's daily lives. Strategies of healthy urban planning and design have been ever more important (165, 166). People's needs, behavior types, and activity patterns in public open spaces were changed correspondingly.

People's physical activity, outdoor leisure activity, and the need for social activity have changed greatly because of the pandemic, and opportunities to adapt to the pandemic conditions differ greatly. For respondents that shifted to working remotely and changed their daily routines, such conditions have led to spending time outdoors more often and for some, spending less time, while others have avoided recreation in crowded areas because of social distancing (167). In particular, the overall volume of physical and social activity decreased greatly, and patterns of specific behavior, such as walking speed, step length, and the number of steps, were found to differ (168). In contrast to the decrease in overall physical and social activities, leisure and recreation activities associated with outdoor space usage increased. Nearly half of the respondents increased outdoor recreation and the majority of outdoor recreation sites were visited more often or as often as before the pandemic (167). Compared with activity levels in 2020 relative to the weather benchmark as a baseline, recreational activity was found to increase greatly by 240% during lockdowns (169). Site preferences also changed accordingly. Among all city land-use zones, a shift was observed in Norway—away from residential and commercial zones toward city green spaces, including forests and protected areas during lockdowns. Moreover, cultural landscapes and protected areas received disproportionately high activity levels relative to the length of recreational trails available within them (169). Regarding specific space usage, spatial analysis in Finland revealed that the most frequently visited recreation sites were near forests, semi-natural areas, and housing areas, as well as places relatively close to the respondents' residences (167). Such impacts were also found to differ according to socio-demographic and urbanization characteristics (170). Among children and adolescents, youth were found to be more affected and worried during the pandemic, and nearly half of the surveyed children and youth reported that changes in their emotional state may contribute to lower subjective well-being (171) although they were more physically active (169). For elders, physical exercise and places closer to home were found to be especially important (172).

**Figure 8:**
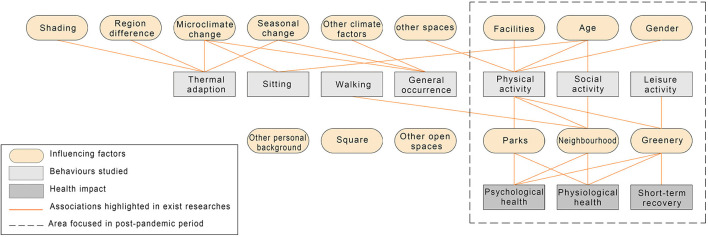
Behavior study in post-pandemic period.

To mitigate behavioral changes caused by COVID-19 and promote physical and psychological health, factors associated with physical activity engagement, social interaction, and perception during outdoor leisure should be further investigated (see Figure 8). Closely associated with public health after the outbreak of COVID-19 (173), the influence of activity-supportive infrastructures such as greenery (102, 174), subjective feeling, and well-being (167, 171) should be examined in more detail. Activities in parks, neighborhoods, and those influenced by facilities should be emphasized. Specific groups' behavioral patterns and needs should receive more attention, among which the influence of age and gender should be considered. Furthermore, although other influencing factors were less related to the physical, leisure, and social attributes of outdoor behavior, their impact in the post-COVID-19 period should not be neglected because of the significant impact of outdoor usage and people's overall occurrence.

## Conclusion

This article is a systematic review focus on behavior-based studies in public open spaces. Followed the method of PRISMA, 109 related research articles were identified. Based on these studies, objective influencing factors' impact were categorized, analyzed, and identified. In specific, distribution of identified papers was first introduced in Section Overview of Identified Papers. Types of behavior being studied, interactions between objective influencing factors and subjective feedbacks were introduced separately. In Section Effects of Objective Influencing Factors, influencing factors' impact were identified. Four types of influencing factors including site location and components, climate-based stimuli, as well as people's background information have been emphasized. Then, health impacts of people's behavior were emphasized, and the research focus in the post-COVID-19 period was further discussed in Section Environmental Constituent.

Several points were highlighted in this research: (1) a relatively complete picture of outdoor influencing factors was proposed; (2) the impacts of factors mainly including the effects of people's background, location and context, environmental components, and environmental stimuli were specified; (3) the distribution of existing papers was analyzed, associations were emphasized, and less studied interactions were distinguished; (4) and research focus after the outbreak of COVID-19 was further discussed.

Moving forward, several points should be considered in future behavior-based investigations: (1) the diverse influencing factors' impact should be more systematically considered in outdoor environments; (2) according to the behavior–factor distribution in existing research, some interactions that are less studied should be considered; (3) factors identified to be associated with outdoor physical, leisure, and social activity promotion and contributing to health benefits should be given more attention in the post-COVID-19 period.

Overall, this paper has provided a relatively systematic review of objective influencing factors frequently studied in existing research. We hope that this paper will benefit design of outdoor built environment and provide a more systematic and complete basis for subsequent behavior-based investigations.

Though most frequently studied influencing factors were extracted and mentioned in this paper, to get a comprehensive understanding of outdoor behavior, further investigation is still needed. As most studies reviewed in this paper were concentrated in regions with better research competence, people's behavior in less developed areas have gained less attention. In addition, subjective factors' impact, such as safety and aesthetics are also highly associated with people's feelings, evaluation, and environmental perception. Such impacts were neglected in this paper while they were also proved to have great impacts of outdoor behavior.

## Data Availability Statement

The original contributions presented in the study are included in the article/supplementary material, further inquiries can be directed to the corresponding author.

## Author Contributions

YY and SH contributed to conception and design of the study. SH and HD organized the database and wrote the first draft of the manuscript. YS, FS, and YZ performed the statistical analysis. DS, XL, HD, SY, and YY wrote sections of the manuscript. All authors contributed to manuscript revision, read, and approved the submitted version.

## Funding

This research is supported by the National Natural Science Foundation of China (Grant No. 52078343, 52078341, and 52078443), and Natural Science Foundation of Shanghai (20ZR1462200). It is also funded by Fundamental Research Funds for the Central Universities (22120210540).

## Conflict of Interest

The authors declare that the research was conducted in the absence of any commercial or financial relationships that could be construed as a potential conflict of interest.

## Publisher's Note

All claims expressed in this article are solely those of the authors and do not necessarily represent those of their affiliated organizations, or those of the publisher, the editors and the reviewers. Any product that may be evaluated in this article, or claim that may be made by its manufacturer, is not guaranteed or endorsed by the publisher.
